# Fueling the fire–a pan-cancer analysis of MYC-regulated lipid metabolism

**DOI:** 10.3389/fcell.2025.1669544

**Published:** 2025-09-24

**Authors:** Tilottama Chatterjee, Ethan Beffert, Daniel Liefwalker

**Affiliations:** ^1^ Department of Biochemistry and Biophysics, Oregon State University, Corvallis, OR, United States; ^2^ Linus Pauling Institute, Oregon State University, Corvallis, OR, United States; ^3^ Environmental and Health Sciences Center, Oregon State University, Corvallis, OR, United States; ^4^ Knight Cancer Institute, Oregon Health and Science University, Portland, OR, United States

**Keywords:** MYC, lipids, metabolism, metabolic reprogramming, fatty acid synthesis, lipid synthesis

## Abstract

The oncogene MYC and its product c-Myc are responsible for a multitude of changes in cancerous cells that trigger cell growth, proliferation and metastasis. The efforts to understand the multifaceted role of MYC in malignancies have highlighted metabolic reprogramming as a prominent function of this transcription factor, with effects across glycolysis, protein and lipid metabolism, mitochondrial respiration and energy storage. In particular, the role of MYC in lipid metabolism has been the focus of several studies in the past two decades, elucidating how the balance of lipid production and breakdown aids in tumor proliferation. Here, we provide a comprehensive summary of how modulation of MYC alters fatty acid synthesis and degradation, the metabolism of compound lipids, and the consequences for other metabolic pathways. The observed effects are highly cell type-specific, highlighting the MYC network’s ability to harness the existing cellular signaling pathways and specific tumor microenvironment to promote tumor growth and metastasis.

## 1 Introduction

The MYC family is comprised of c-MYC (MYC), L-MYC (MYCL) and N-MYC (MYCN), which are proto-oncogenic transcription factors (TF) found to be dysregulated in a broad spectrum of cancers (Jha et al., 2023). Of the three family members, MYC is widely expressed across cell types, and is more prone to oncogenic transformation compared to its paralogs (Schaub et al., 2018). Under non-malignant conditions, tight regulation of MYC expression is maintained by various cellular mechanisms (Dang, 2013; Wahlström and Henriksson, 2015). Oncogenic transformation often involves dysregulated MYC such as upstream activation (Gabay et al., 2014; Kerkhoff et al., 1998), gene amplification (Dalla-Favera et al., 1982; Schaub et al., 2018), translocations (Aukema et al., 2014; Dalla-Favera et al., 1982), or mutations (Malempati et al., 2006; Yeh et al., 2004) that enhance the stability and activity of the MYC protein. Enhanced MYC expression can saturate canonical binding sites leading to recognition of lower affinity sequences (invasion), resulting in genome-wide transcriptional amplification (Lin et al., 2012; Pellanda et al., 2021).

There are significant challenges to targeting MYC activity directly, leading to efforts directed toward inhibiting MYC activity or expression through ancillary methods (McKeown and Bradner, 2014; Whitfield and Soucek, 2025). One of the major downstream effects of MYC overexpression is the metabolic reprogramming of cancer cells. It has been well established that MYC drives increased glycolysis and glutaminolysis along with increased TCA cycling (Goetzman and Prochownik, 2018), and a growing number of studies now discuss the effects of MYC on lipid composition and lipid metabolism in tumor cells. Lipid reprogramming has been shown to affect tumor growth and metastasis, and the cells’ response to therapies. A large proportion of MYC-driven cancer cells show reduced fatty acid oxidation (FAO), and increased fatty acid synthesis (FAS) to provide fatty acids for protein modifications and maintain membrane structure for the consistently proliferating cancer cells (Gouw et al., 2019; Liefwalker et al., 2021; Singh et al., 2021).

The effects of MYC are orchestrated by several cellular cues both upstream and downstream of MYC, which together comprise the MYC network (Carroll et al., 2018; Dang et al., 2006). This network is highly variable depending on tissue type and the effects of the tumor microenvironment. For example, while hepatocytes and fibroblasts both show mitochondrial changes under malignant conditions, these changes are more MYC-reliant for fibroblasts than for hepatocytes (Edmunds et al., 2014). Additionally, MYC inactivation prompts cellular changes that are highly tissue-dependent, as are the effects of MYC reactivation (Gabay et al., 2014). This tissue-specific heterogeneity should be considered when developing therapeutic strategies to target MYC. In this review, we address MYC’s behavior across different cellular models with the aim of summarizing what is currently known about its role in regulating lipid metabolism (Table 1).

**TABLE 1:** Summary of MYC-associated effects across tissue/tumor types.

Tissue/Cells	Cancer	MYC-associated metabolic effects
Lymphocytes	B-cell lymphoma	Acetyl CoA from glycolysis is used for palmitate synthesis; MYC-dependent lipid synthesis is essential for cell survival (Liefwalker et al., 2021) Upregulation of PCYT1A, LPCAT2 in diffuse large B-cell lymphoma (Xiong et al., 2017) Increased serum choline and decreased serum PC in Burkitt’s lymphoma (Yang et al., 2017)
Liver	HB	MYC-dependent upregulation of glycolytic enzymes determines acetyl CoA availability for FAS (Wang et al., 2016)
	HCC	MYC-dependent increases in FAS (Chen et al., 2021; Liefwalker et al., 2021) ACSL4 regulates MYC (Chen et al., 2021) MYC upregulates SREBP1 transcription which controls other liposynthetic enzymes (Gouw et al., 2019) MYC knockout decreases acetyl CoA levels (Wang et al., 2016) Changes in PI and PG levels (Gouw et al., 2019; Shroff et al., 2015)
Pancreas	PDAC	LPP2 controls MYC expression (García-García et al., 2025; Tang et al., 2022) MYC regulates expression of ELOVL1 and ELOVL6 (García-García et al., 2025)
Prostate	PDAC	Upregulation of ACLY, ACC AND FASN (Singh et al., 2021) Downregulation of PLA2G4F, reduced release of arachidonic acid from phospholipids (Whitlock et al., 2022)
Breast	TNBC	Decreased FAS, increased FAO; increased expression of CD36 (Camarda et al., 2016) Decreased expression of FASN and ACC (Casciano et al., 2020)
Lung	Lung adenocarcinoma	MYC increases arachidonate released from phospholipids; reduced levels of PC and PG, which are components of pulmonary surfactants (Hall et al., 2016)
Kidney	Renal cell carcinoma	PG levels affected, specifically: PG (18:1/18:2), PG (18:1/18:1), PG (18:0/18:1), PG (20:4/20:4), PG (18:1/22:6), PG (22:6/22:6), and PG (22:6/22:5) (Shroff et al., 2015)
Oesophagus	Oesophageal squamous cell carcinoma	Overexpression of HMGCR; HMGCR expression was positively associated with cell growth and migration (Zhong et al., 2014)

## 2 Regulation of lipid classes through MYC in various cancers

**FIGURE 1:**
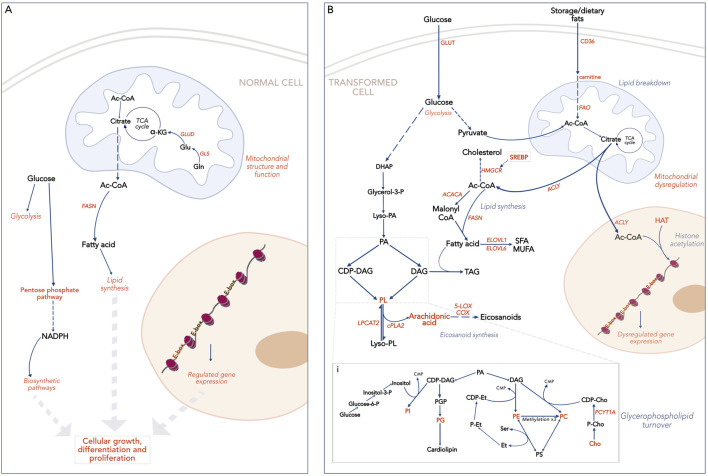
Broad overview of lipid metabolism and associated pathways. Text in orange indicates processes or compounds regulated by MYC. **(A)**. MYC-driven lipid metabolism in a normal cell. Two kinds of E-box sequences are shown: in orange, E-boxes controlled by MYC; in black, E-boxes controlled by other TFs. **(B)**. MYC-driven lipid metabolism in a transformed cell. E-boxes are shown in orange as high levels of MYC lead to genome invasion. i. Glycerophospholipid turnover pathways. HIF-1a, Hypoxia Inducible Factor; E-box, E-box sequence (CAANTG); SREBP, Sterol Regulatory Binding Protein; HMGCR, Hydroxymethylglutaryl CoA reductase; ACC, Acetyl CoA Carboxylase; FASN, Fatty Acid Synthase; SFA, Saturated fatty acids; MUFA, Monounsaturated fatty acids; DHAP, Dihydroxy Acetone Phosphate; PA, Phosphatidic acid; PGP, Phosphatidylglycerol phosphate; PL, glycerophospholipid; DAG, Diacyl glycerol; TAG, Triacyl glycerol; CDP, Cytidyl diphosphate; CMP- Cytidyl monophosphate; P, Phosphate; Et, Ethanolamine; PE, Phosphatidylethanolamine; Cho, Choline; PC, phosphatidylcholine; PS, Phosphatidylserine; PG, Phosphatidylglycerol; HAT, Histone Acetyl Transferase.

In healthy tissues, MYC facilitates cellular development, upregulating pathways that promote cell growth and/or differentiation (Laurenti et al., 2009; Trumpp et al., 2001). This includes anabolic processes like lipid synthesis and the pentose phosphate pathway that provide metabolites required by the expanding cell (Prieto et al., 2021; Stine et al., 2015). Under these conditions, MYC activity is tightly regulated, exerting its effects on target genes through interactions with designated E-box sequences, a motif in gene promoters recognized by MYC and other TFs (Figure 1). Upon dysregulation, MYC’s increased availability causes it to occupy non-MYC E-box sequences as well as its own. Several non-MYC E-box genes, normally under the control of other TFs, are involved in metabolic pathways, either directly or indirectly (Dang, 2012; Kim et al., 2007a). Their MYC-driven expression causes widespread disruption of cellular metabolism.

### 2.1 Lymphoma

MYC-dependent hematopoietic malignancies are driven either by a chromosomal translocation as seen in the case of Burkitt’s lymphoma (Dalla-Favera et al., 1982), or site-specific mutations affecting MYC protein stability and activity (Adhikary et al., 2005). Several studies across B-cell lymphoma models highlight changes in lipid enzymes and metabolites. Acetyl CoA (Ac-CoA) generated from glycolysis was directed towards palmitate synthesis in a MYC-dependent manner, and the lipogenesis pathways were necessary for tumor survival (Liefwalker et al., 2021). Conversely, inhibition of MYCN causes accumulation of lipid droplets due to decreased mitochondrial activity and FAO, suggesting a role for this isoform in regulating fatty acid breakdown (Zirath et al., 2013). Dependency of lymphoid cells on lipid synthesis is a MYC-dependent, but not MYC-exclusive phenomenon. Both RAS and BCR-ABL signaling are known to activate or stabilize MYC activity, so additional studies are needed to understand if MYC regulates lipogenesis in these cancers (Liefwalker et al., 2021).

### 2.2 Liver cancer

As a central hub of energy metabolism, the liver is particularly sensitive to metabolic reprogramming through factors such as MYC. Hepatoblastoma (HB) and hepatocellular carcinoma (HCC) are two liver cancers associated with MYC gene amplification and/or overexpression (Schaub et al., 2018). Although malignant transformation of hepatocytes occurs in both conditions, the difference in MYC dysregulation between HB vs. HCC underscores the importance of MYC in mediating tissue development.

HB is the most prevalent form of pediatric liver cancer, occurring in infants under 3 years of age. MYC does not initiate HB tumorigenesis, but facilitates tumor growth by coordinating energy production pathways (Edmunds et al., 2014). MYC influences lipid metabolism in HB only indirectly, through upregulation of glycolytic enzymes that determine availability of Ac-CoA for fatty acid synthesis (Wang et al., 2016).

MYC is implicated in HCC tumorigenesis and metastasis (Dhanasekaran et al., 2020). Both human and murine HCC cells show increased MYC-dependent lipogenesis, but human HCC cells are resistant to FAS inhibitors (Liefwalker et al., 2021) whereas murine models showed diminished tumor growth following FAS inhibition (Chen et al., 2021). In liver cells, MYC stability is regulated by acyl CoA synthetase, ACSL4. ACSL4 causes MYC dysregulation leading to upregulated SREBP1 transcription and subsequent overexpression of various lipogenesis enzymes (Chen et al., 2021; Gouw et al., 2019). This signaling cascade was further confirmed in clinical HCC samples where a positive association was found between ACSL4 and SREBP1 transcription and cellular triglyceride and cholesterol levels (Gouw et al., 2019). RNA-seq confirmed that HCC tumors exhibit MYC-dependent dysregulation of lipid metabolism genes. Interestingly, recurrent tumors induced by MYC reactivation show different metabolic phenotype from initial tumors, suggesting that the tumor microenvironment influences MYC-dependent metabolic reprogramming (Dolezal et al., 2017).

The specific lipid enzymes upregulated by MYC continue to be a matter of debate, and are likely context dependent. ACLY regulation is cited as MYC-dependent in some cases, although not in HCCs. MYC knockout reduces Ac-CoA levels in HCC cells, suggesting that other enzymes generating or utilizing Ac-CoA are affected by MYC (Wang et al., 2016).

### 2.3 Pancreatic cancer

The rising incidence and poor survival rates of pancreatic ductal adenocarcinoma (PDAC) necessitates the search for therapies that can successfully combat tumor growth and metastasis. The mutations in KRAS is prevalent in 95% of PDAC, and stimulates the MAPK pathway, thereby activating MYC and supporting cancer progression. MYC expression is regulated by lipid phosphate phosphatase (LPP2), an enzyme upregulated in PDAC as well as HCC, breast cancer and melanomas (Chatterjee et al., 2024; Tang et al., 2022). Downstream, MYC binds to the promoters of fatty acid elongases ELOVL1 and ELOVL6 (García-García et al., 2025), upregulating their expression in malignant cells. MYC-dependent transcriptional activity directly upregulates ELOVL6 which supports tumor progression. Inhibition of ELOVL6 results in decreased tumor growth and enhanced uptake of the chemotherapeutic Abraxane.

The transcription factor SREBP1 is known to play an active role in PDAC tumorigenesis through upregulation of lipid metabolizing enzymes (Sunami et al., 2017). As previously described, an association between MYC and SREBP1 has been established in HCC, as well as renal cell carcinoma and T-ALL cells, but further investigation is required to see whether this MYC-dependent interaction holds true in PDAC.

### 2.4 Prostate cancer

Studies in prostate intraepithelial neoplasia and adenocarcinoma demonstrate an overall increase in lipid levels compared to normal prostate tissue. MYC overexpression has been associated with increased fatty acid levels, phospholipids and other metabolites known to be involved in lipid biogenesis (Eberlin et al., 2014; Priolo et al., 2014). Additionally, high MYC mouse models for prostate cancer demonstrate higher circulating levels of both saturated and unsaturated fatty acids, indicating increased synthesis and transport. Presence of MYC was observed at the promoters of key FAS genes ACLY, ACC and FASN (Singh et al., 2021), while it negatively affected phospholipase PLA2G4F which releases arachidonate from phospholipids for prostaglandin production. In agreement with this data, high MYC was also associated with reduced cellular levels of free arachidonic acid which can have significant effects on cellular signaling (Whitlock et al., 2022).

### 2.5 Breast cancer

Several researchers have speculated that a balance of reduced oxidation and increased synthesis of fatty acids is maintained in cancer to supply lipids for growing tumor cells (Broadfield et al., 2021). This pattern, observed in the majority of MYC-focused lipid studies, is contradicted in breast cancer samples which exhibit a trend toward lipid catabolism. It is likely that the adipose-rich tissue of the mammary gland provides an easy source of lipids to the tumor, negating the need to synthesize lipids within their own cells, and allowing more resources to be channeled toward energy production through FAO. Two separate studies have shown an increase in fatty acid oxidation in MYC-dependent triple-negative breast cancer (TNBC). TNBC cells show reduced expression of FASN and ACC2, although no significant difference was observed in ACC1 (Casciano et al., 2020). Additionally, expression of CD36, the plasma membrane fatty acid transporter, was increased, and MYC-dependent cells also showed increased uptake of carnitine, which facilitates entry of fatty acids into the mitochondrial matrix for oxidation. This was accompanied by a relatively modest increase in carnitine palmitoyl transferase, the enzyme that allows transport of carnitine-bound fatty acids for FAO (Camarda et al., 2016).

## 3 Metabolism of lipids derivatives

Several studies have also identified MYC-dependent alterations in the broader lipid network that includes molecules involved in membrane structure, energy storage and cellular signaling. In accordance with its aforementioned association with SREBP1, MYC is also implicated in cholesterol metabolism, inducing the mevalonate pathway through activation of the rate limiting enzyme hydroxymethylglutaryl CoA reductase (HMGCR) (Zhong et al., 2014).

In the case of compound lipids, changes are primarily observed in phospholipid profiles (Figure 1A). The resulting cellular phenotypes are highly variable, depending on the specific needs of a cell type. For example, lung adenocarcinoma cells show a MYC-associated decrease in phosphatidylcholine (PC) and phosphatidylglycerol (PG) which make up pulmonary surfactants crucial to lung tissue functionality. These cells also have increased levels of phosphatidylinositol (PI) and arachidonate-derived phospholipids, which serve as signaling precursors. Inhibition of MYC activity in these cells was associated with a sharp decrease in arachidonic acid and its derivative metabolites (Hall et al., 2016). Changes in arachidonate metabolism have also been observed in prostate cancer and may indicate eicosanoid signaling pathways that are dependent on MYC function (Whitlock et al., 2022).

MYC dysregulates choline metabolism in diffuse large B-cell lymphoma, primarily through upregulation of the phosphate cytidyltransferase choline-a enzyme (PCYT1A), which increases PC synthesis from choline (Xiong et al., 2017). Dysregulated choline metabolism is also observed in Burkitt’s lymphoma, but MYC overexpression is associated with decreased PC levels in these tumors (Yang et al., 2017).

An untargeted lipidomics investigation in lymphoma found that MYC’s effect on PC species was dependent on chain length (Liefwalker et al., 2021). A metabolomics study comparing lymphoma cell lines outlines in detail the differences seen in the lipid profiles of Ras-induced vs. MYC-induced lymphoma, but only covers species in the negative ion mode, accounting for PE and PI, but not PC (Eberlin et al., 2014). A similar exhaustive profiling of PC species would enhance our understanding of choline metabolism in MYC-dependent malignancies, as well as corroborate previous findings from untargeted studies.

MYC is observed to promote PG synthesis across tumor types. Renal cell carcinoma shows no significant MYC-dependent lipid changes, other than variations in certain PG species (Table 1), all of which have also been found upregulated in MYC-dependent lymphoma and HCC (Gouw et al., 2019; Shroff et al., 2015). HCC cells also exhibit changes in PI synthesis in a tissue-specific manner (Gouw et al., 2019).

In some cases, the changes in phospholipids can be correlated directly to the MYC-induced transcriptional changes in phospholipid enzymes (Dolezal et al., 2017; Hall et al., 2016). RNA-seq followed by pathway analysis revealed significant changes in enzymes related to PI metabolism, as well as those involved in bile acid synthesis (Dolezal et al., 2017). Altered choline metabolism was associated with transcriptional activation of PCYT1A, and decreased lysophosphatidylcholine acyl transferase (LPCAT2) (Xiong et al., 2017). Lung tumors with increased signaling lipids showed increased activity of enzyme cPLA2 that releases free fatty acids for conversion to eicosanoids by COX and 5-LOX, which are also upregulated by MYC (Hall et al., 2016).

## 4 How does MYC affect other major metabolic pathways?

MYC’s role in mediating glycolysis and glutaminolysis is well established (Dong et al., 2019; Goetzman and Prochownik, 2018), causing indirect effects on lipid metabolism even where MYC does not directly modulate lipid enzymes. MYC overexpression has been associated with increased utilization of glucose and glutamine (Edmunds et al., 2014), with glutaminase expression being upregulated in prostate cancer (Priolo et al., 2014). Glutaminolysis produces NADPH, an essential cofactor for lipid synthesis (Sunami et al., 2017). Inhibition of glutaminolysis was associated with decreased tumorigenesis and increased longevity of mice with MYC-dependent HCC (Xiong et al., 2017), indicating a high reliance of cancer cells on that pathway.

Regulation of glycolysis occurs at multiple levels-some glycolytic genes (hexokinase II, enolase 1 and lactate dehydrogenase A) bear classical E-box sequences that allow MYC binding (Kim et al., 2004), while activation of hexokinase II and pyruvate dehydrogenase kinase 1 requires cooperation of MYC with HIF-1α (Kim et al., 2007b). MYC also controls glucose transporters, stimulates extracellular export of toxic lactate (Gan et al., 2016) and modulates certain TCA cycle genes (Wahlström and Henriksson, 2015), further optimizing conditions for increased glycolytic flux.

As ever, Ac-CoA’s role as the central metabolic node is critical to understanding how MYC maintains the balance of energy generation and utilization in tumor cells (Figure 1). Tracking Ac-CoA using C_13_-tracing demonstrated that Ac-CoA produced in glycolysis was funneled towards FAS, aided by MYC-dependent enzymes like pyruvate dehydrogenase and Ac-CoA transferase (Liefwalker et al., 2021). Additionally, glutamine metabolism under hypoxic conditions increases pools of Ac-CoA that may also contribute to lipid synthesis (Le et al., 2012).

MYC’s influence on Ac-CoA extends beyond the metabolic realm and encompasses epigenetic changes as well- Ac-CoA generated from citrate by ACLY is used as a substrate for nuclear histone acetylation, which opens up the chromatin to enable gene transcription (Morrish et al., 2008). MYC has been previously shown to enhance histone acetylation through associations with histone acetyl transferase (HAT) complexes (Frank et al., 2003; Kenneth et al., 2007). MYC’s role in histone acetylation may involve careful modulation of Ac-CoA supply and HAT activation; however, investigations into MYC’s effect on ACLY expression and activity have been inconclusive thus far (Singh et al., 2021; Wang et al., 2016).

## 5 Discussion

In the context of a growing and metastasizing tumor, lipid metabolism is important for understanding the sources of energy production and cellular building materials. However, lipids satisfy roles beyond energy production, with eicosanoids as important signaling precursors, and Ac-CoA influencing chromatin regulation. Here, we attempt to summarize the role of the transcription factor c-MYC in regulating different lipid pathways, both directly and indirectly, and what that entails in tumors originating from different tissue types. We find that there is a pattern of increased FAS and decreased FAO observed in a majority of MYC-associated tumors. Some tumors like TNBCs diverge from this pattern (Casciano et al., 2020), and the data on MYC’s effects on certain modified lipids is either inconsistent or incomplete, warranting further investigation into the entire spectrum of metabolites affected in each case. What remains consistent is that MYC tumor progression by 1. Maintaining a constant accelerated growth rate, and 2. Producing enough energy to sustain that growth. MYC-expressing cancer cells generate enough Ac-CoA through increased glucose and glutamine metabolism to meet energy requirements (Edmunds et al., 2014; Le et al., 2012), thus reducing the need for FAO. The surplus metabolites can be channeled toward FAS, which is further facilitated by upregulation of enzymes involved in the pathway. It remains unclear which enzymes are consistently affected by MYC levels, requiring further investigation in future studies.

The majority of studies focus on the role of *c-*MYC, as it is widely distributed across tissues and is the most commonly modified form of the oncogene, but changes in paralogs have also been observed in about a third of all MYC-driven cancers (Schaub et al., 2018). While there have been no observations of MYCL influencing lipid metabolism so far, MYCN appears to affect metabolism in a manner independent of MYC. In prostate cancer, MYCN expression inversely affects expression of ACLY, ACC1 and FASN, unlike MYC which has a positive effect on ACC1 and FASN (Singh et al., 2021). Also contrary to MYC’s effects, neuroblastoma cells show a MYCN-dependent increase in FAO (Dong et al., 2020). MYCN inhibition in fibroblasts was associated with mitochondrial dysfunction and reduced FAO, resulting in accumulation of lipid droplets in the cytosol (Zirath et al., 2013).

The significant advances of the last decade have enabled us to define MYC’s role in metabolic reprogramming, while raising further questions about the intricacies of this regulation, which remain to be resolved. The gaps existing in MYC lipid studies are enhanced by the lack of conclusive and consistent lipidomics data across different experiments. This can be addressed first by the use of techniques like desorption electrospray ionization mass spectrometry imaging (DESI-MSI) which facilitates comprehensive lipid mapping across a cell while minimizing sample loss (Perry, Schroff, Gouw, Eberlin). Additionally, tracing experiments have been successful in determining the metabolic fate of glucose-derived Ac-CoA in palmitate synthesis (Liefwalker et al., 2021) or histone acetylation (Morrish et al., 2010), and can be further implemented in determining the substrates of lipid biogenesis and turnover. Several studies have also successfully incorporated analyses of The Cancer Genome Atlas (TCGA) within their original work, an excellent example of how pre-existing datasets can provide novel insights based on the latest research directions in the field (Camarda et al., 2016; Dhanasekaran et al., 2020; Dolezal et al., 2017; Schaub et al., 2018). Finally, transient MYC expression models–like cells with the Tet-O system allowing conditional expression of MYC (Eberlin et al., 2014; Gouw et al., 2019; Liefwalker et al., 2021; Pajic et al., 2000) – can shed light on the temporal changes associated with MYC modulation, further boosting our understanding of this oncologic target with the goal of developing effective therapeutic strategies.
